# A Degenerate Son

**Published:** 1896-12

**Authors:** 


### EDITORIAL DEPARTMENT.

F. E. DANIEL, M. D., Editor.
S. E. HUDSON, M. D., Managing Editor.
A. J. SMITH. M. D., Galveston, Associate Editor.

EDITORIAL STAFF:

PROF. J. E. THOMPSON, M. D., Texas Medical College, Galveston; Surgery.
PROF. WM. KEILLER, M. D., Texas Medical College, Galveston; Obstetrics and Gynecology.
PROF. DAVID CERNA, M. D., Texas Medical College, Galveston; Therapeutics.
PROF. A. J. SMITH, M. D., Texas Medical College, Galveston; Medicine
DR. R. H. L. BIBB, City of Mexico; Foreign Correspondent.

Published Monthly at Austin, Texas, by Drs. Daniel and Hudson. Subscription price $2.00 a year in advance.

Eastern Agents: The Monihan-Fairchild Special Agency, 24 Park Place, New York City.

Official organ of the West Texas Medical Association, the Houston District Medical Association, the Austin District Medical Society, Brazos Valley Medical Association, the Galveston County Medical Society, and several others

### A DEGENERATE SON.

The trial of Eugene Burt for the murder of his wife and two babies last July, which trial occupied the attention of the (Austin) district court the last week in November, was of more than ordinary interest to physicians, and epecially to the local profession, because the defendant is a son of a distinguished Austin physician, now deceased. Dr. Wm. Jefferson Burt, the father of this man, was once Secretary of the State Medical Association, and will be remembered by all who ever met him, as a genial, pleasant and courteous gentlemen. He was prominent at his home, socially and professionally, and was universally respected as a man, exemplary in every relation of life. That his son should be capable of such a crime excites surprise, and the fact can only be attributed to insanity;—there is no other theory at all plausible, and no motive known. Insanity is hereditary; circumstances developed it in young Burt,—the first symptoms manifesting themselves on the death of his father, which occurred when this boy was sixteen years of age, just entering upon the physiological change or development of the sexual system—a period at which, it is well known, any predisposition to insanity is apt to develop; a fact, by the bye, overlooked by the defense. A long train of unfortunate circumstances, calculated to contribute to the development of latent insanity occurred, and the morbid mental action, it seems, culminated in an explosion—or uncontrollable impulse, during which the crime was committed. Having exhausted all resources for money—having no means, no friends, harrassed by debts, in fear of arrest for forgery, no employment, and turned out of the house his family occupied, they would have been in a state of extreme destitution; and it is not improbable, in the absence of a better conjecture, or evidence of a motive, that he slayed them *to save them from want;* killed them because he loved them; and evidence showed that he was devoted to them.

But the testimony of the experts, Drs. Wallace, Worsham, three doctors Wooten, Davis, Smith and Graves—a preponderance of the testimony taken, convinced the jury that the defendant was of **sound mind*, or "sane," at the time of the killing, and he was accordingly convicted and sentenced to death. Only the testimony of Drs. Swearingen and McLaughlin was at all favorable to the views we have above expressed. Dr. Swearingen, State Health Officer, gave it as his opinion in knowledge of all the facts, that the man was insane at the time of the act; that it was an explosion of insanity. In this line Dr. McLaughlin followed somewhat; giving his opinion, that Burt is, and was, morallv insane; all agreeing that he is a "moral pervert." Dr. Tally, of Temple, testified to insanity in the family, to his knowledge, and believes the man is a degenerate, i. e., a congenital criminal, and ergo, morally insane. Dr. Worsham testified, however, that moral insanity is no longer recognized. Doctors will differ. Dr. Worsham is Superintendent of the State Lunatic Asylum at Austin; Dr. Wallace, of Waco, is an ex-superintendent of large experience.

The case furnished a topic of much interest to the Austin doctors, and caused many of them to brush up on insanity.

It is an interesting psychological study, and finds many parallels in the recent works on criminal anthropology; characteristics of the case, as elicited at the trial, being well known as those of the †natural criminal (insane)—the born, incurable

---

*True, Dr. Wallace gave it as his opinion that Burt was of unsound mind, but "not insane in the sense in which insanity is understood," a distinction which we fail to appreciate.—Ed.]

†An insane criminal is one who commits a crime and becomes insane afterwards; a criminal insane — or congenital criminal degenerate (Lombroso) or congenital (insane) delinquent (Ferri) is one, insane from birth and insane at the time of the killing.

criminal-degenerate, or delinquent; and enumerated as distinctive features, in the works of Ferri, Lombroso, Havelock-Ellis and others.

The death of Burt at the hands of the law will be "murder most foul."

